# FOXO transcriptional activity is associated with response to chemoradiation in EAC

**DOI:** 10.1186/s12967-022-03376-w

**Published:** 2022-04-25

**Authors:** A. Creemers, A. P. van der Zalm, A. van de Stolpe, L. Holtzer, M. Stoffels, G. K. J. Hooijer, E. A. Ebbing, H. van Ooijen, A. G. C. van Brussel, E. M. G. Aussems-Custers, M. I. van Berge Henegouwen, M. C. C. M. Hulshof, J. J. G. H. M. Bergman, S. L. Meijer, M. F. Bijlsma, H. W. M. van Laarhoven

**Affiliations:** 1 Laboratory for Experimental Oncology and Radiobiology, Center for Experimental and Molecular Medicine, Cancer Center Amsterdam, Amsterdam UMC, University of Amsterdam, Amsterdam, The Netherlands; 2 Department of Medical Oncology, Cancer Center Amsterdam, Amsterdam UMC, University of Amsterdam, Meibergdreef 9, 1105 AZ Amsterdam, The Netherlands; 3 Philips Molecular Pathway Diagnostics, Eindhoven, The Netherlands; 4 Philips Research, Eindhoven, The Netherlands; 5 Department of Pathology, Cancer Center Amsterdam, Amsterdam UMC, University of Amsterdam, Amsterdam, The Netherlands; 6 Department of Surgery, Cancer Center Amsterdam, Amsterdam UMC, University of Amsterdam, Amsterdam, The Netherlands; 7 Department of Radiotherapy, Cancer Center Amsterdam, Amsterdam UMC, University of Amsterdam, Amsterdam, The Netherlands; 8 Department of Gastroenterology, Cancer Center Amsterdam, Amsterdam UMC, University of Amsterdam, Amsterdam, The Netherlands; 9 Oncode Institute, Amsterdam, the Netherlands

**Keywords:** Esophageal adenocarcinoma, Pathway analysis, Predictive, Neoadjuvant chemoradiation therapy

## Abstract

In this study we aimed to investigate signaling pathways that drive therapy resistance in esophageal adenocarcinoma (EAC). Paraffin-embedded material was analyzed in two patient cohorts: (i) 236 EAC patients with a primary tumor biopsy and corresponding post neoadjuvant chemoradiotherapy (nCRT) resection; (ii) 66 EAC patients with resection and corresponding recurrence. Activity of six key cancer-related signaling pathways was inferred using the Bayesian inference method. When assessing pre- and post-nCRT samples, lower FOXO transcriptional activity was observed in poor nCRT responders compared to good nCRT responders (*p* = 0.0017). This poor responder profile was preserved in recurrences compared to matched resections (*p* = 0.0007). PI3K pathway activity, inversely linked with FOXO activity, was higher in CRT poor responder cell lines compared to CRT good responders. Poor CRT responder cell lines could be sensitized to CRT using PI3K inhibitors. To conclude, by using a novel method to measure signaling pathway activity on clinically available material, we identified an association of low FOXO transcriptional activity with poor response to nCRT. Targeting this pathway sensitized cells for nCRT, underlining its feasibility to select appropriate targeted therapies.

**Supplementary Information:**

The online version contains supplementary material available at 10.1186/s12967-022-03376-w.

## Introduction

Over the past three decades, incidence rates of esophageal adenocarcinoma (EAC) have been progressively increasing [1]. This increase is up to six fold in Western countries and still rising, characterized by a striking rise in incidence of males. For patients with locoregional EAC disease the preferred treatment strategy is neoadjuvant chemoradiation therapy (nCRT) or perioperative chemotherapy followed by an esophagectomy [2], but prognosis remains poor [3]. Thus far, no targeted treatment options are available in the curative setting. Treatment strategies directed against signaling pathways that drive treatment resistance, could improve therapy outcomes.

EAC is characterized by a complex network of aberrant signal transduction pathways, making therapeutic targeting challenging [4, 5]. In order to develop novel targeting strategies, these aberrant pathways must be identified. Up until now, methods to examine such pathways have relied on assessments of protein (over-)expression or activation, copy number variations, gene mutations, and full transcriptome through RNA-sequencing approaches [6]. However, these modalities have limited value for implementation in clinical practice. The fresh frozen tissue required for many of these analyses is usually not available, and often the expression of a single protein is assessed, not fully capturing relevant tumor biology. Here, we address these issues by a novel previously described computational Bayesian approach to interpret mRNA expression levels of gene sets [6–8]. Target gene mRNA expression levels are used to infer transcription complex activity of the corresponding signal transduction pathway and thereby the activity of the signal transduction pathway. The advantage of such analysis is that a more complete and therefore reliable read-out of the signal transduction pathway is obtained, compared to an mRNA measurement as a proxy for merely its corresponding protein activity [6, 7]. Importantly, mRNA extracted from a small amount of formaldehyde fixed-paraffin embedded (FFPE) tissue, which is readily available in clinical practice, suffices for the analysis.

The aim of this study was to identify aberrant signaling pathways that drive therapy resistance in EAC. To reveal candidate signaling pathways as targets for targeted therapy, we investigated key signal transduction pathways in material available from daily clinical routine. Tissue specimens before and after nCRT, and for primary tumor and recurrent disease were assessed and compared to clinicopathological outcome data. Using patient-derived cell lines, candidate signaling pathways were targeted to study their potential therapeutic relevance.

## Material and methods

### Patient selection

Medical records of a national referral center for esophagogastric cancer, the Amsterdam UMC, location Academic Medical Center, were systematically searched for patients with histologically proven EAC, including gastric esophageal junction (GEJ), treated between June 2004 and May 2013. (i) Resectable disease: patients treated with curative intent by an esophagectomy as single treatment or by the CROSS regimen: nCRT with paclitaxel (50 mg/m^2^ body-surface area), carboplatin (AUC 2 ml/min), 41.4 Gy radiotherapy in 23 fractions of 1.8 Gy, followed by resection. As part of a randomized phase II study [9], panitumumab, an anti-EGFR monoclonal antibody, was added to the nCRT regimen. Sensitivity analysis showed no significant differences between nCRT only or nCRT + panitumumab, thus in following analysis groups were merged. Cases with available pre-treatment biopsy of the primary tumor site and the corresponding resection specimen were selected. (ii) Recurrent disease: patients with available resection specimen of the primary tumor site and a corresponding metachronous recurrence. Medical records were extracted by a trained physician using standardized data extraction concerning the location of primary tumor, TNM stage of pathological resections reports (pTNM), Mandard response score, received treatment and survival. Confirmation of adenocarcinoma histological subtype, response to therapy and non-cancerous healthy tissue were assessed by a trained pathologist.

### RNA analysis

Using hematoxylin and eosin (H&E) stained slides of included biopsies and resection specimens, FFPE tumor blocks containing the highest tumor percentage were selected by a trained pathologist. Selected H&E slides were scanned using Philips Ultra-Fast Scanner (UFS; Philips, Eindhoven, the Netherlands). Tumor areas were marked by a trained pathologist using the Digital Pathology Images portal (Philips, Eindhoven, The Netherlands). A minimal area of 2 mm^2^ was annotated. In case of diffuse tumor distribution, areas with a high tumor density were selected. Using a custom-built device, digital annotations of whole slide H&E scans were transferred to an adjacent H-stained slide and deparaffinized. Marked tumor areas were scraped off for RNA extraction with the RNeasy kit (Qiagen). qPCR data from extracted RNA were subjected to stringent quality checks (QC) prior to determining signaling pathway activity scores of the androgen receptor (AR), estrogen receptor (ER), Phosphatidylinositol 3 Kinase (PI3K)-Forkhead Box O (FOXO), Hedgehog (HH), Transforming growth factor receptor Beta (TGF-β) and Wingless-Integrated (WNT) pathways by applying the computational model described below [6, 7, 10–12].

### Measuring activity of signal transduction pathway activity

Measuring signal transduction pathway activity on Affymetrix U133 Plus2.0 data has been described extensively before. Pathway activity was inferred from target gene mRNA levels of six key oncogenic signaling pathways that play a role in tumor growth and metastasis [6] using a Bayesian network, inferring the odds for pathway activity, as published earlier [6, 7, 10, 11]. Affymetrix assays were converted to qPCR-based assays, developed and performed by Philips (Philips Molecular Pathway Dx, Eindhoven) [13]. Samples that failed quality control (QC) were removed from analysis. PI3K pathway activity is inversely related to the measured FOXO transcription factor activity, on the premise that no cellular oxidative stress is present. Therefore, FOXO activity score was interpreted together with SOD2 target gene expression level to distinguish between oxidative stress- and growth control-induced FOXO activity, as described before [7, 11].

### Cell culture and treatment

Primary EAC cell lines EAC007B, EAC031M, EAC058M, EAC081R and EAC289B were established from patient EAC material as previously described [14]. Primary cell lines EAC007B and EAC031M were cultured in Advanced DMEM/F12 (Gibco) supplemented with N2 (5 ml; Invitrogen), HEPES (5 mM; Life Technologies), D-glucose (0.15%; Sigma-Aldrich), β-mercaptoethanol (100 μM; Sigma-Aldrich), Insulin (10 μg/ml; Sigma-Aldrich), Heparin (2 μg/ml; Sigma-Aldrich) and 1:1000 Trace elements B and C (Fisher Scientific) [15], EAC058M, EAC081R and EAC289B were cultured in DMEM (Gibco). Publicly available EAC cell lines Flo1 (RRID:CVCL_2045), OE19 (RRID:CVCL_1622) and OE33 (RRID:CVCL_1622; ATCC, Manassas, VA) were maintained in RPMI (Lonza). All cell lines have been authenticated using STR profiling within a year. All experiments were performed with mycoplasma-free cells. All medium during experiments contained 2% fetal bovine serum, L-glutamine (2 mM; Sigma-Aldrich), penicillin and streptomycin (500 µg/mL; Lonza). Carboplatin and paclitaxel were purchased from the Amsterdam UMC clinical pharmacy, as used for EAC patients in nCRT setting. The nCRT regimen was mimicked in vitro by challenging all cell lines 7 days with CRT, comprising carboplatin (20 μM) and paclitaxel (0.05 nM) combined with 1 Gy radiation daily as described earlier [16]. On day 1 cells were plated, on day 2–5, cells received 1 Gy radiation per day and from day 2–7 cells were exposed to chemotherapy and PI3K inhibitors LY3023414, Alpelisib, Pictilisib or Idelalisib (Additional file 5: Table S2). Assays were performed on day 8. An Axiovert 200 M microscope (Zeiss) was used to obtain phase contrast images.

### Western blot

Pre-treatment cells were lysed in RIPA buffer (Cell Signaling) containing phosphatase and protease inhibitor cocktail (Cell Signaling). Protein levels were determined by BCA (Pierce). Samples were heated for 5 min at 95 ºC loaded on 4–20% polyacrylamide precast gels (Bio-Rad) and transferred to PVDF membranes. Samples were blocked with 5% BSA (Lonza) in Tris buffered saline with 0.1% Tween-20 (TBS-T), and incubated overnight at 4 °C with primary antibodies (Additional file 3: Table S3). All were used at 1:1000. (HRP)-conjugated secondary were used at 1:5000 and incubated for 2 h at room temperature. Proteins were imaged using a FuijFilm LAS 4000 imager (Fuji), using ECL Western blotting substrate (Pierce). Western blot bands were quantified using Image J by dividing protein of interest per lane by the housekeeping protein.

### Cell viability assay

Cell viability was determined using a Cell Titer-Blue Cell Viability Assay kit (G8081; Promega, Madison, WI). Cells were seeded into 96-well plates in triplicates. After cell adhesion overnight, cells were treated. After one week, cell viability was measured by adding 20 μL of Cell Titer-Blue reagent to each well followed by three hours incubation. Plates were read at 560/590 nm in a cytofluormeter (BioTek Instruments). Viability was calculated from values from CRT cells with or without PI3K inhibitors, minus baseline cell viability.

### Imaging based proliferation assay

Proliferation was determined using IncuCyte™ live cell imaging system (Essen BioScience), quantitatively detecting live cells. Cells were imaged after one week of treatment.

### Apoptosis assay

Apoptosis was similarly assessed using the IncuCyte system, by incubating cells in 0.33 mg/mL annexin V-FITC, administered simultaneously with drugs. Cells were imaged after one week of treatment. Apoptotic fraction was calculated by the ratio of FITC-positive cell area, divided by confluence (total cell area).

### Gene expression database analysis

Gene expression of Broad Hallmark *PI3K_AKT_mTor_signaling* gene set was correlated with the Broad Hallmark *Epithelial_Mesenchymal_transition* gene set in two publicly available datasets: Esophageal Adenocarcinoma Fitzgerald (GSE96669)[17] and Esophageal Carcinoma Tumor Cancer Genome Atlas (TCGA-ESCA; https://gdc-portal.nci.nih.gov/projects/TCGA-ESCA) [4]. Analysis was performed using the web‐based genomics platform R2 (R2: Genomics Analysis and Visualization Platform, http://r2.amc.nl).

### Statistical analyses

Pathway activity between normal and EAC tissue were calculated using the Mann–Whitney test to compare ranks. In case of paired samples—between matched pre-treatment biopsies and resection specimen or resection specimen and recurrence, the Wilcoxon matched-pairs signed ranked test was used. Correlation of pathway activities was assessed using Pearson’s correlations. Sensitivity analyses were performed for patients receiving panitumumab [9]. Survival analyses were performed using Kaplan–Meier and multivariable Cox proportional hazard regression analysis, including clinically relevant clinicopathological variables. Statistical analyses were performed in R. A p-value of p < 0.05 was regarded statistically significant. In all in vitro experiments Spearman correlation tests were performed using GraphPad Prism 8. Error bars in bar graphs indicate the mean ± SD. A *p-*value of p < 0.05 was considered statistically significant.

## Results

### Study population selection

**Fig. 1:**
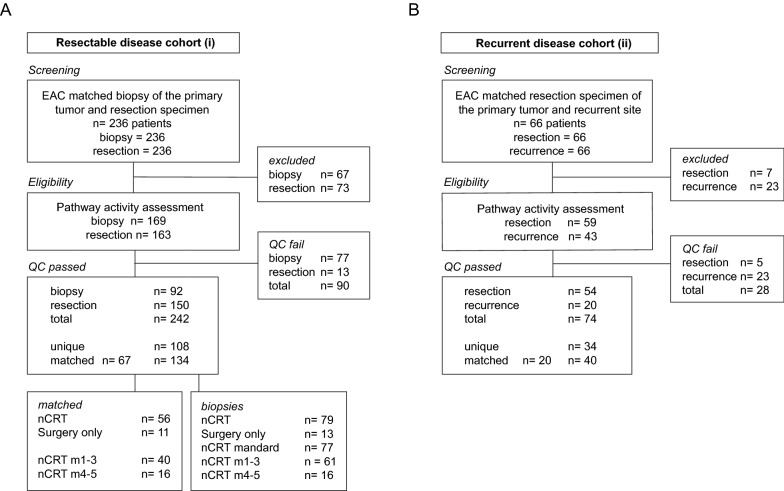
Patient selection. **A** Selection of matched pre-treatment biopsies and post treatment resection specimens of EAC patients in the Amsterdam UMC between June 2004 and May 2013. Samples were excluded due to availability or insufficient tumor tissue (shown in right panel). Included samples in lower panels. The screening panel indicates the number of patients, in subsequent other panels the numbers represent number of specimens. **B** Selection of matched resected primary tumor site and metachronous recurrence of EAC patients, as in A. QC: quality control

In order to identify aberrant signaling pathways that drive therapy resistance in EAC, clinically obtained material from the Amsterdam UMC that was stored in FFPE was retrospectively screened for eligibility. Two EAC patient cohorts were assembled: a resectable disease cohort (i), and a recurrent disease cohort (ii). For the resectable disease cohort (i), 236 EAC cases with matched biopsies of the primary tumor site and post-nCRT resection specimens were identified (Fig. 1A). The majority of patients was male (N = 154 of 181, 85.1%, Table 1) and presented with advanced stage disease (T3–4, N = 154, 85.1%). nCRT was given in 175 cases (83.4%). A complete pathological response (pCR, Mandard score 1, no residual tumor) was attained in 11 (6.3%) of the 175 nCRT treated patients. For analysis also tissue samples with very low or no residual tumor cells were taken along as the tumor microenvironment plays an essential role in EAC therapy resistance [12]. Recurrence was detected in 52.5% (N = 92) of the patients. Median annotated tumor area of included biopsies was 9.4 mm^2^ and 38.4 mm^2^ for resection specimens. In total, 67 biopsies and 73 resection specimens were excluded due to reasons of availability or insufficient tissue and could therefore not be processed for pathway analysis. After stringent quality control, 242 specimens of 181 patients were included, comprising 92 biopsies and 150 resections. Biopsy and resection could be matched for 67 patients, rendering 134 matched and 108 unique samples. Of the 92 biopsies, 79 later received nCRT, of which 77 a mandard score could be determined after surgery.

**Table 1:** Patient characteristcs of the resectable (i) and recurrent (ii) cohort

	Resectable cohort (i)		Recurrent cohort (ii)	
	n = 236	100%	n = 66	100%
Mean age at dianosis	57.6	(36–81)	64.7	(40–85)
Sex				
Male	200	84.7	51	77.3
Female	36	15.3	15	22.7
Location				
Proximal	1	0.4	0	0.0
Mid	1	0.4	3	4.5
Distal	58	24.6	41	62.1
GEJ	176	74.6	22	33.3
T-stage				
1	5	2.1	3	4.5
2	35	14.8	5	7.6
3	192	81.4	57	86.4
4	4	1.7	1	1.5
N-stage				
0	75	31.8	16	24.2
1	140	59.3	41	62.1
2	1	0.4	7	10.6
3	20	8.5	2	3.0
Treatment				
nCRT	172	72.9	15	22.7
nCRT + P	27	11.4	2	3.0
CRT + HT	0	0.0	1	1.5
CT	0	0.0	1	1.5
Surgery only	37	15.7	47	71.2
Mandard grade				
1	26	11.0	0	0.0
2	38	16.1	1	1.5
3	77	32.6	10	15.2
4	40	16.9	6	9.1
5	18	7.6	1	1.5
NA	37	15.7	48	72.7
Recurrence	128	54.2	66	100.0
Alive	75	31.8	5	7.6

Similar clinicalpathological distributions observed in both cohorts, P = panitumumab

Mandard grade NA due to no nCRT, surgery only

Matched material of resected primary tumor site and metachronous recurrence for the recurrent disease cohort (ii), was available for 66 patients (Fig. 1B). Seven resections and 23 recurrences were excluded due to insufficient tissue. After QC, 74 specimens of 53 patients remained, comprising 54 resections and 20 recurrences. Resection and recurrence could be matched in 20 patients, rendering 40 matched and 34 unique samples. Of the 53 included patients, 39 patients underwent surgery without prior treatment (73.6%; Table 1). The remaining 15 patients (27.8%) received neoadjuvant treatment prior to resection of the esophagus. The majority of patients was male (N = 41 of 53, 77.4%), had tumors located in the distal esophagus (N = 33, 62.3%) and pT-advanced staged disease (T3–4 N = 50, 94.4%). Median annotated tumor area of included resections was 144.7 mm^2^ and 20.9 mm^2^ for recurrences.

### Low FOXO transcriptional activity associates with poor response to nCRT

**Fig. 2:**
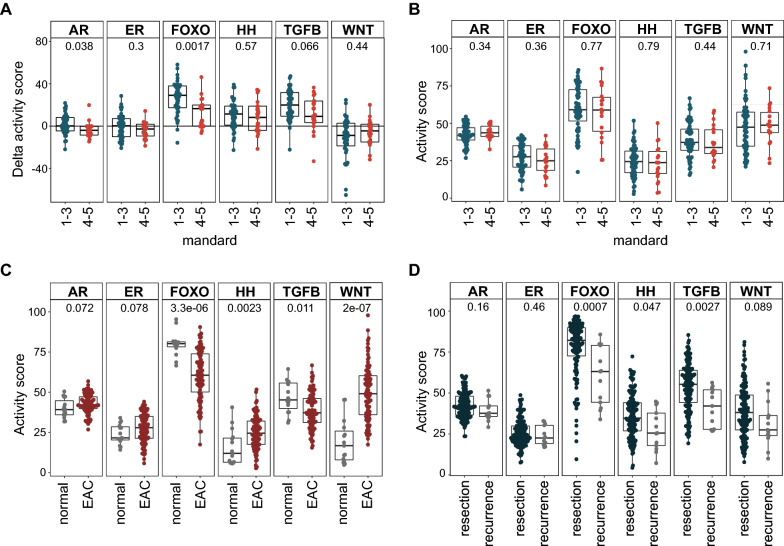
Low FOXO activity corresponds with a poor nCRT responder phenotype in EAC patient samples. **A** Activity of six key signal transduction pathways was measured in the resectable disease cohort (i) and compared to clinicopathological outcome data. Shown pathways from left to right: androgen receptor (AR), estrogen receptor (ER), Phosphatidylinositol 3-Kinase/Forkhead Box O (FOXO), Hedgehog (HH), Transforming growth factor receptor Beta (TGF-β) and Wnt pathway. Pre-treatment biopsy pathway activity scores were subtracted from matched post-nCRT resections (N = 56), yielding a *delta* activity score. Unique samples that passed QC were excluded from analysis. Two-sided Wilcoxon signed-rank statistical tests were performed between Mandard low (1–3, N = 40) and Mandard high (4–5, N = 16) patients per pathway. **B** Pathway activity scores of pre-treatment EAC biopsies in the recurrent disease cohort with known Mandard score (N = 77). Both matched and unique samples were included for analysis. Wilcoxon statistical tests compare Mandard low (1–3, N = 61) and Mandard high (4–5, N = 16) patients per pathway. **C** Pathway activity scores of non-treated EAC resection tissue from both the resected and recurrent disease cohort (N = 37 + 47 = 84) and adjacent healthy esophageal tissue (N = 20). **D** Pathway signal transduction activity was measured in the recurrent disease cohort (ii). Pathway activity scores of all resected specimens (N = 150) and recurrences (N = 20) that passed QC are shown. *p-*values are indicated in the figures. Boxplots represent median with interquartile range

In order to reveal candidate signaling pathways in EAC patients associated with response to nCRT, pathway activity of the primary tumor before and after nCRT was assessed in the resectable disease cohort (i). Pathway signal transduction activity of six key oncogenic signal transduction pathways [6] was measured and compared to clinicopathological outcome data. Comparing pre-treatment biopsies and post-nCRT resection specimens (i.e. resections minus biopsies), FOXO transcriptional activity was found to remain lower in poor nCRT responders compared to good nCRT responders (Mandard 4–5 vs 1–3, *p* = 0.002, respectively, Fig. 2A). Both AR and TGF-β pathway pathway activities remained lower in these groups as well, albeit not statistically significant (TGF-β) or with a small difference between the medians (AR) (AR: *p* = 0.038, TGF-β: *p* = 0.066). These data show that patients with a worse pathological response to nCRT have persistently lower FOXO activity in both biopsy and resection specimens compared to patients with a good response to nCRT. Unfortunately, EAC pre-treatment biopsies showed no significant differences in activity of the assessed pathways (Fig. 2B). When comparing pre-treatment biopsies and post-nCRT resections, FOXO (*p* < 1∙10^–3^), TGF-β (*p* < 1∙10^–3^) and HH (*p* = 0.003) activities were increased after nCRT, compared to no difference or a small decrease in patients who received surgery only (Additional file 1: Fig. S1A). This shows that the nCRT regimen rather than progression over time influenced pathway activity scores.

Although tumor tissue is characterized by high activation of oncogenic signal transduction pathways, which pathways is cancer type specific. To assess if any pathway activity scores from EAC tumor tissue are differentially expressed from the healthy tissue from which it is derived, non-treated EAC tissue (N = 84) was compared to adjacent normal healthy esophageal tissue (N = 20). EAC tumor tissue was characterized by lower FOXO activity (*p* < 3.3∙10^–6^; Fig. 2C). This was accompanied by higher WNT, HH and lower TGF-β pathway activity (*p* = 2∙10^–7^, *p* = 0.0023, and *p* = 0.011, respectively).

As FOXO and TGF-β pathway activities often showed the same trend between groups, we investigated whether these two pathways were correlated with each other. When exclusively examining post-nCRT resection specimens, FOXO and TGF-β activity were highly correlated in Mandard high (4–5) patients, while this was not the case in Mandard low (1–2) patients, supporting the hypothesis that combined low activity of both FOXO and TGF-β pathway after nCRT is related to poor prognosis (Mandard low R = 0.47, *p* = 0.24, Mandard high R = 0.73, *p* < 1∙10^–8^, Additional file 1: Fig.S1B). Although a similar correlation was observed in pre-treatment samples, this was less evident and not associated with pathological response (Mandard low R = 0.11, *p* = 0.7, Mandard high R = 0.52, *p* = 0.039, Additional file 1: Fig.S1C). Moreover, a numerically lower median disease-free survival of 17 versus 97 months was seen in patients with combined low versus combined high pathway activities in post-nCRT resections (cut-off by median pathway activity scores FOXO, 82.72; TGF-β, 56.32; *p* = 0.055) (Additional file 1: Fig.S1D). Hence, in EAC patients combined low FOXO and TGF-β pathway activity after nCRT in resection specimens is associated with poor prognosis.

Next, to examine if a similar pathway activity profile could be observed in resection versus recurrent EAC specimens, we investigated the recurrent cohort (ii). In patients who had recurrent disease after nCRT and esophagectomy, reduced combined FOXO and TGF-β activity was seen in recurrent sites compared to matched resection specimens (FOXO *p* = 0.0007 and TGF-β *p* = 0.0027, respectively, Fig. 2D). This implies that low combined FOXO and TGF-β pathway activity is a profile associated with recurrent disease which is further accentuated when the disease progresses.

In conclusion, in patient subsets with a poor response to nCRT and patients with recurrent disease, we consistently found combined low activity of FOXO transcription factors, and the TGF-β pathway. Therefore, targeting these pathways additionally to nCRT could possibly improve clinical outcome in poor responders. Of all individual pathways, FOXO activity scores showed the highest discriminative power for the response to nCRT (Fig. 2A and D). Additionally, as the net contributions of tumor-suppressive and tumor-promoting actions of TGF-β are currently not fully understood in the context of EAC [18], we decided to assess whether targeting the PI3K-FOXO pathway could have a beneficial effect in chemoradiation treatment.

### PI3K-FOXO pathway can be targeted to improve CRT response in EAC cell lines

**Fig. 3:**
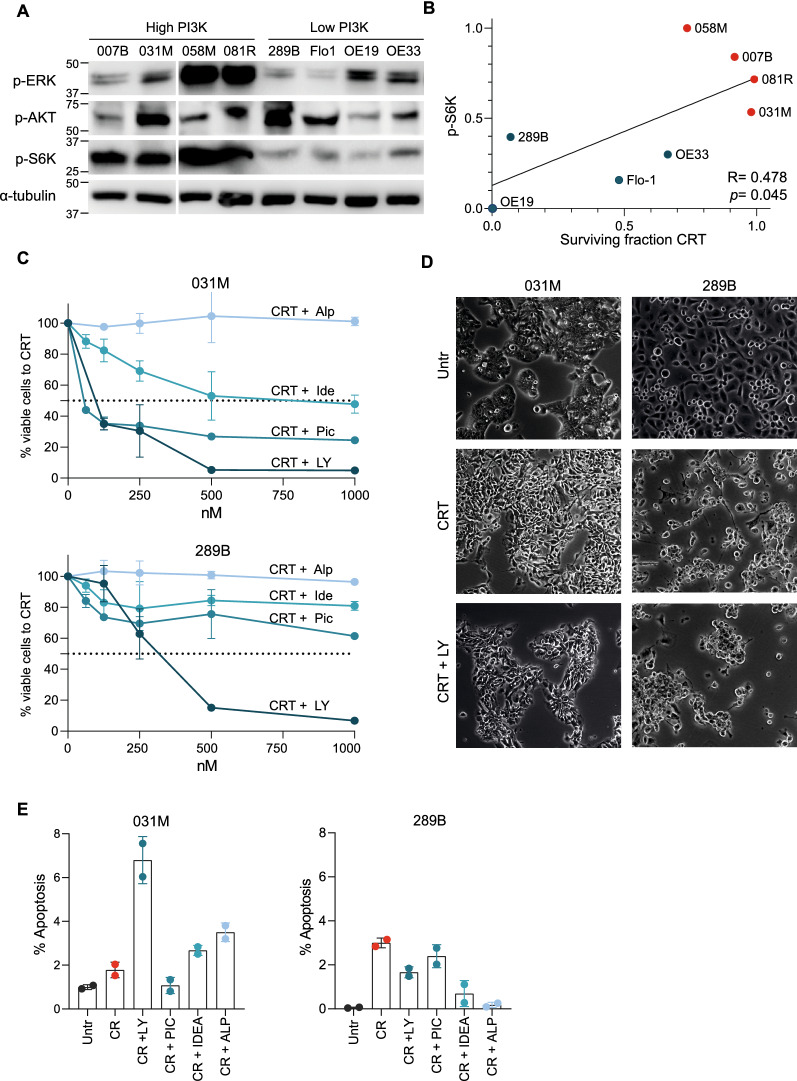
Targeting PI3K-FOXO pathway sensitizes EAC cells to CRT. **A** Western blot analysis of the PI3K-FOXO pathway in eight untreated EAC cell lines, assessed by phospho-AKT, phospho-ERK and phospho-S6K. α-tubulin was used as loading control. The membrane was sliced to exclude an ESC sample, indicated by the break. **B** Poor CRT responder cell line 031 M and good CRT responder cell line 289B were treated for 7 days with CRT, surviving fraction was measured on day 8. Graph shows Spearman correlation of surviving fraction after CRT versus quantified p-S6K corrected to α-tubulin as in (**A**). **C** Cells were treated for 7 days with the CRT regimen, including a concentration range of 0, 62.5, 125, 250, 500 and 1000 nM Alpelisib, Idelalisib, Pictilisib or LY3023414 [26–31]. Percentage viable cells were measured on day 8 and plotted normalized to CRT. Data represents two biological replicates with SEM. **D** Morphological analyses of poor responder 031 M and good responder 289B cells in untreated conditions, 7 days nCRT and 7 days nCRT + LY3023414 with 500 nM. **E** Poor CRT responder cell line 031 M and good responder cell line 289B were treated for 7 days with the CRT regimen in addition to 500 nM of PI3K inhibitors (based on average IC50 of four compounds). Apoptosis measured by percentage of green fluorescent Annexin V-FITC. Datapoints represent biological replicates with SD

FOXO transcription factors are known to be under negative control by the PI3K pathway, by activity of kinases AKT and S6 [19–23] and are well-known to be inversely correlated to PI3K signaling [21, 24, 25]. To investigate whether PI3K pathway inhibitors could have a beneficial additive effect in combination with nCRT, a panel of eight EAC cell lines was used, of which five derived from EAC patients treated at our hospital (EAC007B, EAC031M, EAC058M, EAC081R, EAC289B) and three publicly available EAC cell lines (Flo1, OE19, OE33). Cell lines were both assessed for FOXO transcriptional activity based on the Bayesian method, as well as baseline PI3K pathway activity by Western blot for phospho-ERK, phospho-AKT and phospho-S6 Kinase (pS6K) (Fig. 3A). When baseline FOXO transcriptional activity from these cell lines was correlated to PI3K pathway protein activity, no significant association could be found. (Additional file 2: Fig. S2A). We decided to continue with PI3K pathway assessment by Western blot, allowing a direct assessment of phospho pathway activity in vitro. Subsequently, these cell lines were exposed to our previously established in vitro CRT regimen [16] and the surviving fraction after CRT was measured. In line with our patient data, high PI3K pathway activity determined by pS6K, was associated with poor response to CRT in vitro. pS6K was found to most strongly correlate with response to CRT (Fig. 3B; R = 0.478, *p* = 0.045). High pS6K cell lines were relatively resistant to CRT (EAC007B, EAC031M, EAC058M and EAC081R), whereas low pS6K cell lines were more sensitive (289B, OE19, OE33 and OE33). We take these data to indicate that in vitro, pre-treatment PI3K pathway activity as determined by pS6K, associates with, and potentially drives poor response to CRT.

Aiming to improve anti-tumor efficacy of CRT in poor responder cell lines, the additive effect of PI3K inhibition to the CRT regimen was assessed. Four PI3K inhibitors with positive Phase I clinical results were selected; LY3023414, Alpelisib, Pictilisib and Idelalisib (Additional file 5: Table S2). First, primary cell lines EAC031M (poor CRT responder) and 289B (good CRT responder) were exposed to CRT alone, or with PI3K inhibitors to validate inhibition of the PI3K pathway. Indeed, combined CRT with PI3K inhibition resulted in lower PI3K pathway activity determined by pS6K, in which the poor-responder cell line EAC031M demonstrated a more pronounced response (Additional file 2: Fig.S2B, C). Next, the full panel of cell lines was exposed to a concentration range of PI3K inhibitors and CRT. Of note, a higher sensitivity to PI3K inhibitors was seen in the poor CRT responder cell line EAC031M, compared to the good CRT responder cell line 289B (Fig. 3C). Additional cell lines showed that poor CRT responder cell lines (EAC007B, EAC058M and EAC081R) as well had a higher sensitivity to PI3K inhibitors. (Additional file 2: Fig.S2D). PI3K inhibitor LY3023414 demonstrated the highest potency to inhibit cell viability in nearly all cell lines tested.

In patient-derived EAC cell lines, our group has previously shown CRT to induce epithelial-to-mesenchymal transition (EMT), characterized by a mesenchymal cellular morphology and therapy resistance [16]. Indeed, the PI3K signature was strongly associated with EMT gene expression programs in publicly available data sets (R = 0.554, *p* < 0.000 in GSE96669 [17], and R = 0.273 *p* = 0.009 in the EAC samples in TCGA-ESCA [4]; Additional file 3: Fig.S3A). In concordance, the addition of LY023414 to CRT resulted in a reduction of mesenchymal cell morphology induced by CRT (Fig. 3D) and EMT marker expression (CXCR4) [32], most notably in poor responder EAC031M cells (Additional file 3: Fig. S3B). This demonstrates that PI3K inhibition in poor CRT responder cell lines can serve to thwart resistance mechanisms possibly by counteracting mesenchymal tumor cell programs. To further demonstrate that combined targeting of PI3K with CRT results in cell death rather than merely reduced cell growth, we measured apoptosis in poor- and good-CRT responder cell lines. Poor responder cell lines such as EAC031M showed the most robust increase in apoptosis when exposed to PI3K inhibitors LY3023414, Idelalisib and Alpelisib (Fig. 3E, additional cell lines in Additional file 4: Fig. S4A). To conclude, these exploratory results indicate that Phase I PI3K inhibitors could be a promising addition to chemoradiation regimens, but the successful application of such inhibitors hinges on patient selection based on baseline PI3K-FOXO pathway activity.

## Discussion

Using clinically available FFPE material we were able to determine activity of key cancer driving pathways before and after nCRT using small amounts of EAC tissue. Persistent low FOXO transcriptional activity was associated with poor response to nCRT in EAC patient samples. This poor nCRT responder profile was also seen in recurrences of nCRT-pretreated patients. In addition, heterogeneity in high PI3K activity, inversely linked with FOXO, was seen in patient-derived cell lines, providing a valuable tool to experimentally target candidate signaling pathways, such as PI3K-FOXO. Our exploratory data demonstrate that PI3K inhibition can indeed sensitize nCRT-resistant cell lines.

As EAC is known to be a heterogeneous tumor, the full characteristics of the tumor might not be represented by the analysis of single tumor sites. This could be problematic, as large intratumoral heterogeneity might hamper patient selection for targeted therapy. In addition, not merely tumor cells but also stromal and immune cells influence EAC tumor biology [15]. These environmental stimuli are only partially captured in our analyses. Moreover, a potential lack in our study is the absence of post-treatment healthy esophageal tissue, to compare with pre-treatment healthy tissue as a control for treatment effects of nCRT on FOXO or other transcriptional activities. Additionally, as merely one FFPE tissue slide was used, sometimes insufficient mRNA was extracted in good nCRT responders (post-nCRT Mandard 1 score). By using two or more FFPE slides, this issue could be circumvented. Nevertheless, we demonstrate that in a single tissue sample from a possibly heterogeneous and effectively treated tumor, clinically relevant sample characteristics can be determined.

Here we described that low FOXO transcriptional activity is associated with worse clinical outcome, sometimes combined with low TGF-β pathway activity. TGF-β signaling has a complex dual role in human cancer [18, 33]. Via the canonical Smad pathway TGF-β signaling has tumor suppressive effects in early carcinomas. As tumors develop, these protective effects of TGF-β are often lost and TGF-β signaling switches to promote cancer progression, as we have shown before using flow cytometry analysis [16]. Nevertheless, based on our mRNA based pathway activity analysis TGF-β signalling activity in EAC samples seems to be predominantly tumor suppressive. Given that the dual role of TGF-β has not been fully elucidated in EAC, to design a new treatment strategy, we focused on targeting the PI3K-FOXO pathway.

The phosphatidylinositol 3-kinase (PI3K) pathway is one of the main cellular growth factor signaling pathways frequently hyperactivated in cancer [34]. Because PI3K pathway activity negatively regulates forkhead box-O (FOXO) transcription factor activity, FOXO target gene expression is inversely correlated with PI3K activity. Additionally, numerous studies have revealed that high PI3K and low FOXO activity is associated with a poor prognosis [25, 35, 36], although little research has been done in EAC [35, 36]. As our results show an association with poor response and metastatic behavior, in line with other cancer types, PI3K inhibition appears a promising candidate for therapeutic targeting in EAC patients [10, 37].

To experimentally test novel therapeutic strategies as PI3K pathway inhibition, preclinical cancer models representative of patients’ tumor biology are essential. We found that patient-derived cell lines show heterogeneity in PI3K pathway activity, allowing their use as a tool to test predictive signals for response to PI3K pathway inhibition. In line with our patient data, both good and poor nCRT responder cell lines were identified with corresponding PI3K pathway activity on phosphorylated protein level. In the relatively small number of cell lines we could not find and association of nCRT on FOXO transcriptional activity level. However, as phosphorylation plays an essential role in activation of the comprehensive PI3K pathway, we were able to detect a relation on protein phosphorylation level. Although these experimental data are encouraging, caution must be taken drawing conclusions as additional experiments should be performed in 3D models.

Nonetheless, we identified a PI3K inhibitor that could give a beneficial additional effect to nCRT; LY3023414, a novel PI3K/mTOR inhibitor, with potent activity in EAC in rat models [38]. Interestingly, LY3023414 demonstrated a manageable safety profile in a phase 2 clinical trial [39], as improved clinical responses of all four used inhibitors [26–29, 39, 40]. Hence, we believe it is of value to investigate the use of PI3K pathway inhibition in patients with EAC, either combined with nCRT, or as adjuvant therapy.

To conclude, we were able to determine the activity of key cancer driving pathways before and after nCRT in clinically attainable amounts of EAC tissue. The poor nCRT responder profile was detected in tissue of the primary site, recurrences of nCRT-pretreated patients and patient derived cell lines. Hence, the pathway activity model described here may be used to identify patients irresponsive to nCRT and select for appropriate targeted therapies.

### Novelty and impact

By using a novel Bayesian inference method to measure signaling pathway activity on clinically available material, we identified an association of low FOXO transcriptional activity with poor response to nCRT. Targeting this pathway sensitized cells for nCRT, underlining its feasibility to select appropriate targeted therapies.

## Supplementary Information

**Additional file 1: Figure S1.** Poor responder phenotype in patient samples. **A** Pathway signal transduction activity of six key signal transduction pathways was measured in the resectable disease cohort (i). Pre-treatment biopsy pathway activity scores were subtracted from all matched resections that both passed QC, i.e. delta activity score (N = 69). Two-sided Wilcoxon signed-rank statistical tests were performed between all post-nCRT (N = 56) and surgery only (N = 13) patients. *p-*values are indicated in the figures. Boxplots represent median with interquartile range. **B** All post-nCRT resection specimens from the resectable disease cohort were assessed for correlations between FOXO and TGF-β activity. Spearman correlations were performed, N = 138, separated for low (1–2, N = 17, all Mandard 2), middle (3, N = 59) and high (4–5, N = 47) Mandard score. **C** Pre-treatment biopsies were assessed for correlations between FOXO and TGF-β activity. Spearman correlations were performed, N = 77, separated for low (1–2, N = 22), middle (3, N = 37) and high (4–5, N = 18) Mandard score obtained after nCRT. **D** Disease free survival of patients with combined low FOXO and TGF-β pathway activity versus combined high pathway activities in post-nCRT resection specimens (N = 83). Cut-off by median pathway activity score.

**Additional file 2: Figure S2.** Validation of PI3K pathway inhibition and sensitization of poor CRT responder cells. **A** Correlation of baseline FOXO transcriptional activity with PI3K pathway activity based on P-S6, P-AKT and P-ERK PI3K in all eight EAC cell lines. **B** Poor CRT responder cell line 031 M and good CRT responder cell line 289B were exposed for 7 days to the CRT regimen in combination with 500 nM LY3023414, Alpelisib, Pictilisib or Idelalisib. Cells were lysed on day 8. Western blot analysis of PI3K-FOXO pathway by P-AKT and P-S6K as proteins of interest, β-actin as loading control. **C** Quantification of Western blot in A corrected for α-tubulin. **D** Cells were treated for 7 days with the CRT regimen, including a concentration range of 0, 62.5, 125, 250, 500 and 1000 nM Alpelisib, Idelalisib, Pictilisib or LY3023414. Percentage viable cells were measured on day 8 and plotted normalized to CRT. Data represents two biological replicates with SEM.

**Additional file 3: Figure S3.** PI3K inhibitors can revert CRT-induced EMT. **A** Correlation of gene expression of Broad Hallmark *Pi3K_AKT_mTor_signalling* gene set with the Broad Hallmark *Epithelial_Mesenchymal_transition* gene set in two publicly available datasets, GSE96669 and Esophageal Carcinoma Tumor Cancer Genome Atlas (TCGA-ESCA), respectively. **B** FACS analyses of mesenchymal marker CXCR4 after 7 days of treatment with CRT with or without 500 nM LY3023414. 031 M poor CRT responder, 289B good CRT responder. gMFI = geometric mean fluorescent intensity.

**Additional file 4: Figure S4.** Apoptosis induced by PI3K pathway inhibitors in poor responder CRT cell lines. Poor CRT responder cell lines 007B, 058 M and 081R and good responder cell lines Flo1, OE19 and OE33 were treated for 7 days with the CRT regimen in addition to 500 nM of PI3K pathway inhibitors (based on average IC50 of four compounds). Apoptosis measured by percentage of green fluorescent Annexin V-FITC. Data points represent biological replicates, mean with SD.

**Additional file 5: Table S1.** Characteristics of the five established patient derived cell lines. **Table S2.** FDA approved PI3K inhibitors tested in this study. **Table S3** Western blot antibodies used in this study.

## Data Availability

The data that support the findings of this study are available from ArrayExpress. Restrictions apply to the availability of these data, which were used under license for this study. Data are available with the permission of Philips Research.

## Abbreviations

AR: Androgen receptor
CROSS: Chemo Radiotherapy for Oesophageal cancer followed by Surgery Study
ER: Estrogen receptor
EAC: Esophageal adenocarcinoma
HH: Hedgehog
nCRT: Neoadjuvant chemoradiotherapy
PI3K-FOXO: Phosphatidylinositol 3 Kinase (PI3K)-Forkhead Box O
TGF-β: Transforming growth factor receptor Beta
WNT: Wingless-Integrated

## References

[1] Coleman HG, Xie S-H, Lagergren J (2017). The epidemiology of esophageal adenocercinoma. Gastroenterology.

[2] Lordick F, Mariette C, Haustermans K, Obermannová R, Arnold D, ESMO Guidelines Committee (2016). Oesophageal cancer ESMO clinical practice guidelines for diagnosis, treatment and follow-up. Ann Oncol.

[3] Sjoquist KM, Burmeister BH, Smithers BM, Zalcberg JR, Simes RJ, Barbour A (2011). Survival after neoadjuvant chemotherapy or chemoradiotherapy for resectable oesophageal carcinoma: an updated meta-analysis. Lancet Oncol.

[4] Network CGAR (2017). Integrated genomic characterization of oesophageal carcinoma. Nature.

[5] Frankell AM, Jammula S, Li X, Contino G, Killcoyne S, Abbas S (2019). The landscape of selection in 551 esophageal adenocarcinomas defines genomic biomarkers for the clinic. Nat Genet.

[6] Verhaegh W, van Ooijen H, Inda MA, Hatzis P, Versteeg R, Smid M (2014). Selection of personalized patient therapy through the use of knowledge-based computational models that identify tumor-driving signal transduction pathways. Cancer Res.

[7] van Ooijen H, Hornsveld M, Dam-de Veen C, Velter R, Dou M, Verhaegh W (2018). Assessment of functional phosphatidylinositol 3-kinase pathway activity in cancer tissue using Forkhead Box O target gene expression in a knowledge-based computational model. Am J Pathol.

[8] Stolpe AV, Holtzer L, van Ooijen H, de Inda MA, Verhaegh W (2019). Enabling precision medicine by unravelling disease pathophysiology: quantifying signal transduction pathway activity across cell and tissue types. Sci Rep.

[9] Kordes S, van Berge Henegouwen MI, Hulshof MC, Bergman JJ, van der Vliet HJ, Kapiteijn E (2014). Preoperative chemoradiation therapy in combination with panitumumab for patients with resectable esophageal cancer: the PACT study. Int J Radiat Oncol Biol Phys.

[10] van de Stolpe A (2019). Quantitative measurement of functional activity of the PI3K signaling pathway in cancer. Cancers (Basel).

[11] van de Stolpe A, Holtzer L, van Ooijen H, Inda MA, Verhaegh W (2019). Enabling precision medicine by unravelling disease pathophysiology: quantifying signal transduction pathway activity across cell and tissue types. Sci Rep.

[12] Verhaegh W, Van de Stolpe A (2014). Knowledge-based computational models. Oncotarget.

[13] Inda MA (2020). Estrogen receptor pathway activity score to predict clinical response or resistance to neoadjuvant endocrine therapy in primary breast cancer. Mol Cancer Ther.

[14] Damhofer H, Ebbing EA, Steins A, Welling L, Tol JA, Krishnadath KK (2015). Establishment of patient-derived xenograft models and cell lines for malignancies of the upper gastrointestinal tract. J Transl Med.

[15] Ebbing EA, van der Zalm AP (2018). Stromal-derived interleukin 6 drives epithelial-to-mesenchymal transition and therapy resistance in esophageal adenocarcinoma. Proc Natl Acad Sci.

[16] Steins A, Ebbing EA, Creemers A, van der Zalm AP, Jibodh RA, Waasdorp C, Meijer SL, van Delden OM, Krishnadath KK, Hulshof M, Bennink RJ, Punt CJA, Medema JP, Bijlsma MF, van Laarhoven HWM (2019). Chemoradiation induces epithelial-to-mesenchymal transition in esophageal adenocarcinoma. Int J Cancer.

[17] Bornschein J, Wernisch L, Secrier M, Miremadi A, Perner J, MacRae S (2019). Transcriptomic profiling reveals three molecular phenotypes of adenocarcinoma at the gastroesophageal junction. Int J Cancer.

[18] Lebrun JJ (2012). The dual role of TGFbeta in human cancer: from tumor suppression to cancer metastasis. ISRN Mol Biol.

[19] Brunet A, Bonni A, Zigmond MJ, Lin MZ, Juo P, Hu LS (1999). Akt promotes cell survival by phosphorylating and inhibiting a Forkhead transcription factor. Cell.

[20] Czech MP (2003). Insulin's expanding control of forkheads. Proc Natl Acad Sci USA.

[21] Seoane J, Le HV, Shen L, Anderson SA, Massague J (2004). Integration of Smad and forkhead pathways in the control of neuroepithelial and glioblastoma cell proliferation. Cell.

[22] Tran H, Brunet A, Griffith EC, Greenberg ME (2003). The many forks in FOXO's road. Sci STKE.

[23] Vivanco I, Sawyers CL (2002). The phosphatidylinositol 3-Kinase AKT pathway in human cancer. Nat Rev Cancer.

[24] Yao S, Fan LY, Lam EW (2018). The FOXO3-FOXM1 axis: A key cancer drug target and a modulator of cancer drug resistance. Semin Cancer Biol.

[25] Santo EE, Stroeken P, Sluis PV, Koster J, Versteeg R, Westerhout EM (2013). FOXO3a is a major target of inactivation by PI3K/AKT signaling in aggressive neuroblastoma. Cancer Res.

[26] Bendell JC, Varghese AM, Hyman DM, Bauer TM, Pant S, Callies S, Lin J, Martinez R, Wickremsinhe E, Fink A, Wacheck V (2018). A first-in-human phase 1 study of LY3023414, an oral PI3K/mTOR dual inhibitor, in patients with advanced cancer. Clin Cancer Res.

[27] Zaidi AH, Kosovec JE, Matsui D, Omstead AN, Raj M, Rao RR, Biederman RW, Finley GG, Landreneau RJ, Kelly RJ, Jobe BA (2017). PI3K/mTOR dual inhibitor, LY3023414, demonstrates potent antitumor efficacy against esophageal adenocarcinoma in a rat model. Ann Surg.

[28] Elkabets M, Pazarentzos E, Juric D, Sheng Q, Pelossof RA, Brook S, Benzaken AO, Rodon J, Morse N, Yan JJ, Liu M, Das R, Chen Y, Tam A, Wang H, Liang J, Gurski JM, Kerr DA, Rosell R, Teixidó C, Huang A, Ghossein RA, Rosen N, Bivona TG, Scaltriti M, Baselga J (2015). AXL mediates resistance to PI3Kα inhibition by activating the EGFR/PKC/mTOR axis in head and neck and esophageal squamous cell carcinomas. Cancer Cell.

[29] Soria JC, Adjei AA, Bahleda R, Besse B, Ferte C, Planchard D, Zhou J, Ware J, Morrissey K, Shankar G, Lin W (2017). A phase IB dose-escalation study of the safety and pharmacokinetics of pictilisib in combination with either paclitaxel and carboplatin (with or without bevacizumab) or pemetrexed and cisplatin (with or without bevacizumab) in patients with advanced non-small cell lung cancer. Eur J Cancer.

[30] Schöffski P, Cresta S, Mayer IA, Wildiers H, Damian S, Gendreau S, Rooney I, Morrissey KM, Spoerke JM, Ng VW, Singel SM (2018). A phase Ib study of pictilisib (GDC-0941) in combination with paclitaxel, with and without bevacizumab or trastuzumab, and with letrozole in advanced breast cancer. Breast Cancer Res.

[31] Borazanci E, Pishvaian MJ, Nemunaitis J, Weekes C, Huang J, Rajakumaraswamy N (2020). A phase Ib study of single-agent idelalisib followed by idelalisib in combination with chemotherapy in patients with metastatic pancreatic ductal adenocarcinoma. Oncologist.

[32] Yuanqiang Lin QM, Li Lin, Wang Hui (2018). The CXCL12–CXCR4 axis promotes migration, invasiveness, and EMT in human papillary thyroid carcinoma B-CPAP cells. Biochem Cell Biol.

[33] Syed V (2016). TGF-β signaling in cancer. J Cell Biochem.

[34] Vogelstein B, Papadopoulos N, Velculescu VE, Zhou S, Diaz LA, Kinzler KW (2013). Cancer genome landscapes. Science..

[35] Yin X, Feng C, Han L, Ma Y, Jiao Y, Wang J, Jia L, Jing F, Gao X, Zhang Y, Zhang J (2018). Diallyl disulfide inhibits the metastasis of type II esophagealgastric junction adenocarcinoma cells via NF-κB and PI3K/AKT signaling pathways in vitro. Oncol Rep.

[36] Kresty LA, Weh KM, Zeyzus-Johns B, Perez LN, Howell AB (2015). Cranberry proanthocyanidins inhibit esophageal adenocarcinoma in vitro and in vivo through pleiotropic cell death induction and PI3K/AKT/mTOR inactivation. Oncotarget.

[37] Cedres S, Montero MA, Martinez P, Martinez A, Rodriguez-Freixinos V, Torrejon D (2012). Exploratory analysis of activation of PTEN-PI3K pathway and downstream proteins in malignant pleural mesothelioma (MPM). Lung Cancer.

[38] Zaidi AH, Kosovec JE, Matsui D, Omstead AN, Raj M, Rao RR (2017). PI3K/mTOR dual inhibitor, LY3023414, demonstrates potent antitumor efficacy against esophageal adenocarcinoma in a rat model. Ann Surg.

[39] Rubinstein MM, Hyman DM, Caird I, Won H, Soldan K, Seier K (2020). Phase 2 study of LY3023414 in patients with advanced endometrial cancer harboring activating mutations in the PI3K pathway. Cancer.

[40] Andre F, Ciruelos E, Rubovszky G, Campone M, Loibl S, Rugo HS (2019). Alpelisib for PIK3CA-mutated, hormone receptor-positive advanced breast cancer. N Engl J Med.

