# Supplementary figures and images for: FOXO transcriptional activity is associated with response to chemoradiation in EAC
Source: J Transl Med. 2022 Apr 25;20:183. doi: 10.1186/s12967-022-03376-w (PMC9036728; doi:10.1186/s12967-022-03376-w)

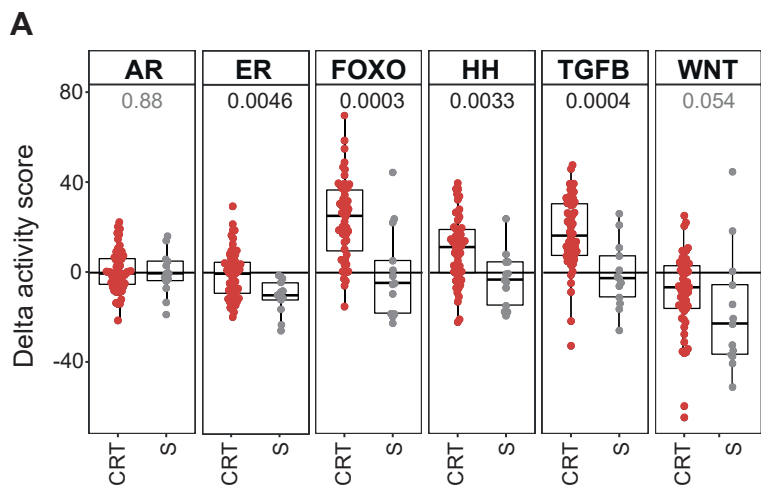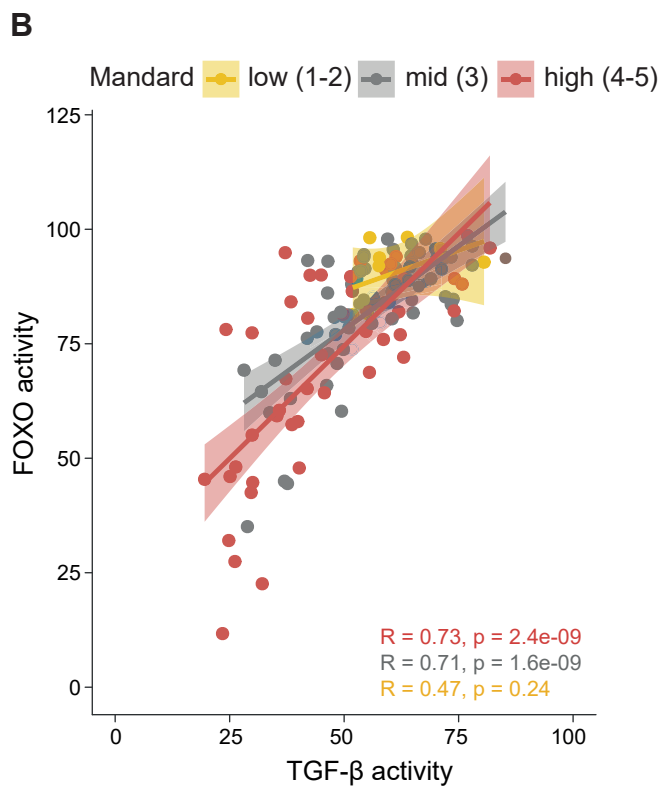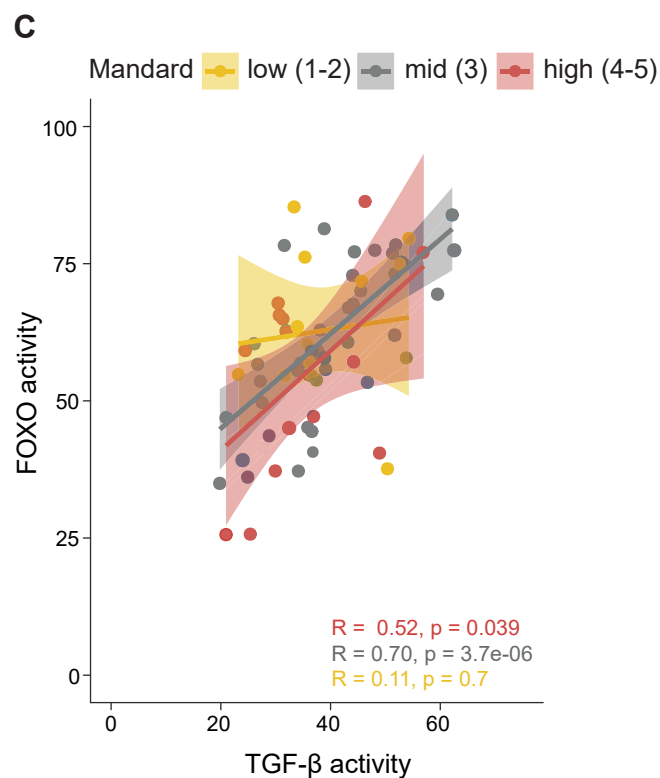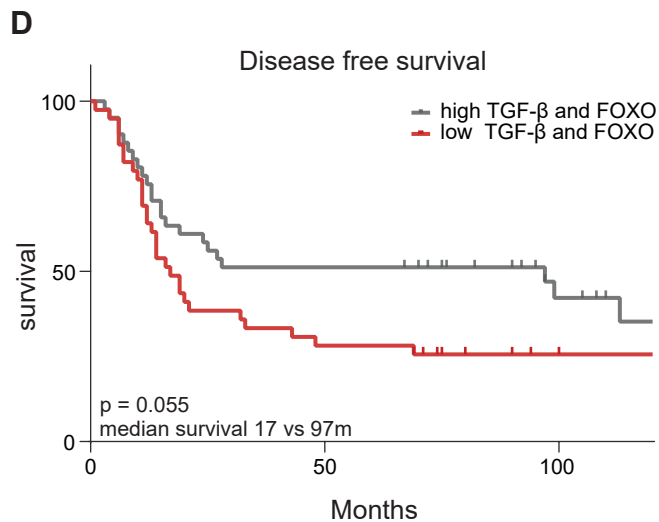

Supplement: Supplementary file 1 — Additional file 1: Figure S1. Poor responder phenotype in patient samples. A Pathway signal transduction activity of six key signal transduction pathways was measured in the resectable disease cohort (i). Pre-treatment biopsy pathway activity scores were subtracted from all matched resections that both passed QC, i.e. delta activity score (N = 69). Two-sided Wilcoxon signed-rank statistical tests were performed between all post-nCRT (N = 56) and surgery only (N = 13) patients. p-values are indicated in the figures. Boxplots represent median with interquartile range. B All post-nCRT resection specimens from the resectable disease cohort were assessed for correlations between FOXO and TGF-β activity. Spearman correlations were performed, N = 138, separated for low (1–2, N = 17, all Mandard 2), middle (3, N = 59) and high (4–5, N = 47) Mandard score. C Pre-treatment biopsies were assessed for correlations between FOXO and TGF-β activity. Spearman correlations were performed, N = 77, separated for low (1–2, N = 22), middle (3, N = 37) and high (4–5, N = 18) Mandard score obtained after nCRT. D Disease free survival of patients with combined low FOXO and TGF-β pathway activity versus combined high pathway activities in post-nCRT resection specimens (N = 83). Cut-off by median pathway activity score. [file 12967_2022_3376_MOESM1_ESM.pdf]

Supplementary figure 2

**A**

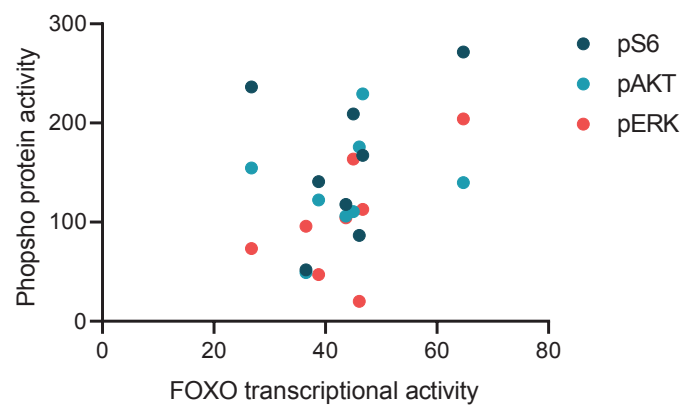

**B**

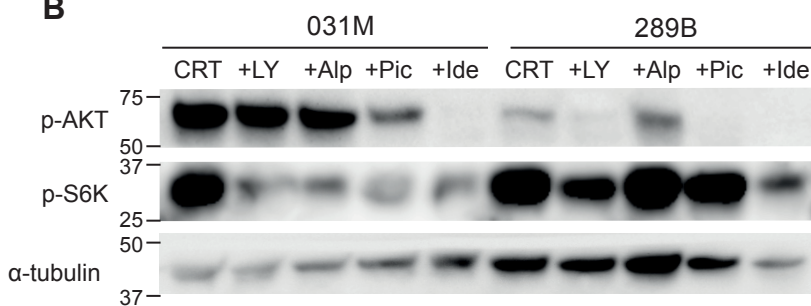

**C**

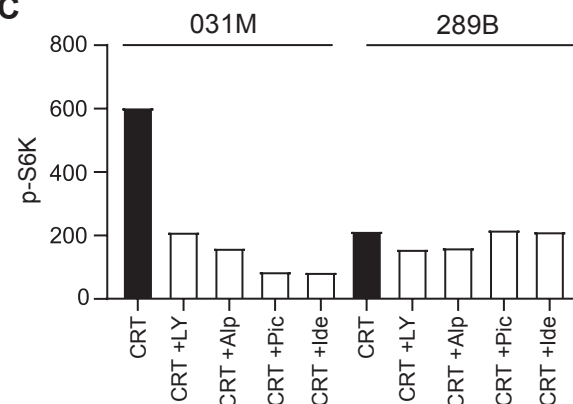

**D**

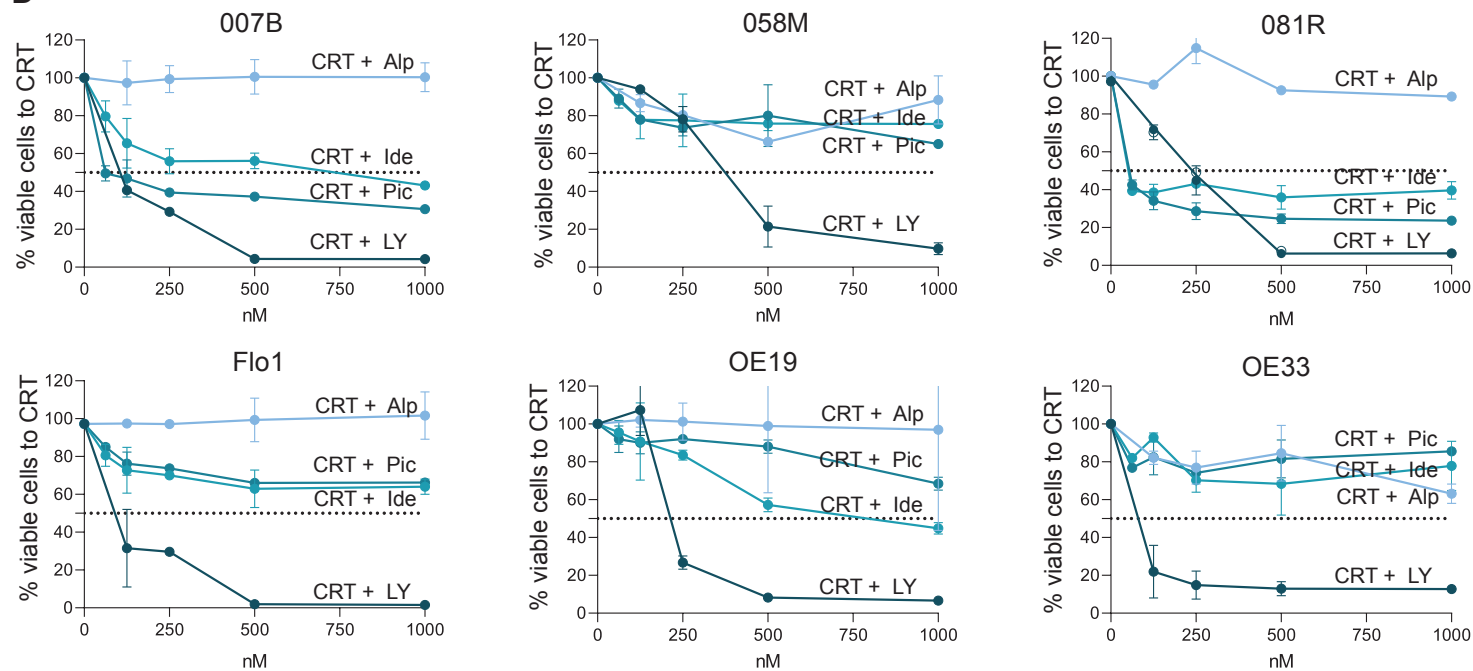

Supplement: Supplementary file 2 — Additional file 2: Figure S2. Validation of PI3K pathway inhibition and sensitization of poor CRT responder cells. A Correlation of baseline FOXO transcriptional activity with PI3K pathway activity based on P-S6, P-AKT and P-ERK PI3K in all eight EAC cell lines. B Poor CRT responder cell line 031 M and good CRT responder cell line 289B were exposed for 7 days to the CRT regimen in combination with 500 nM LY3023414, Alpelisib, Pictilisib or Idelalisib. Cells were lysed on day 8. Western blot analysis of PI3K-FOXO pathway by P-AKT and P-S6K as proteins of interest, β-actin as loading control. C Quantification of Western blot in A corrected for α-tubulin. D Cells were treated for 7 days with the CRT regimen, including a concentration range of 0, 62.5, 125, 250, 500 and 1000 nM Alpelisib, Idelalisib, Pictilisib or LY3023414. Percentage viable cells were measured on day 8 and plotted normalized to CRT. Data represents two biological replicates with SEM. [file 12967_2022_3376_MOESM2_ESM.pdf]

Supplementary figure 3

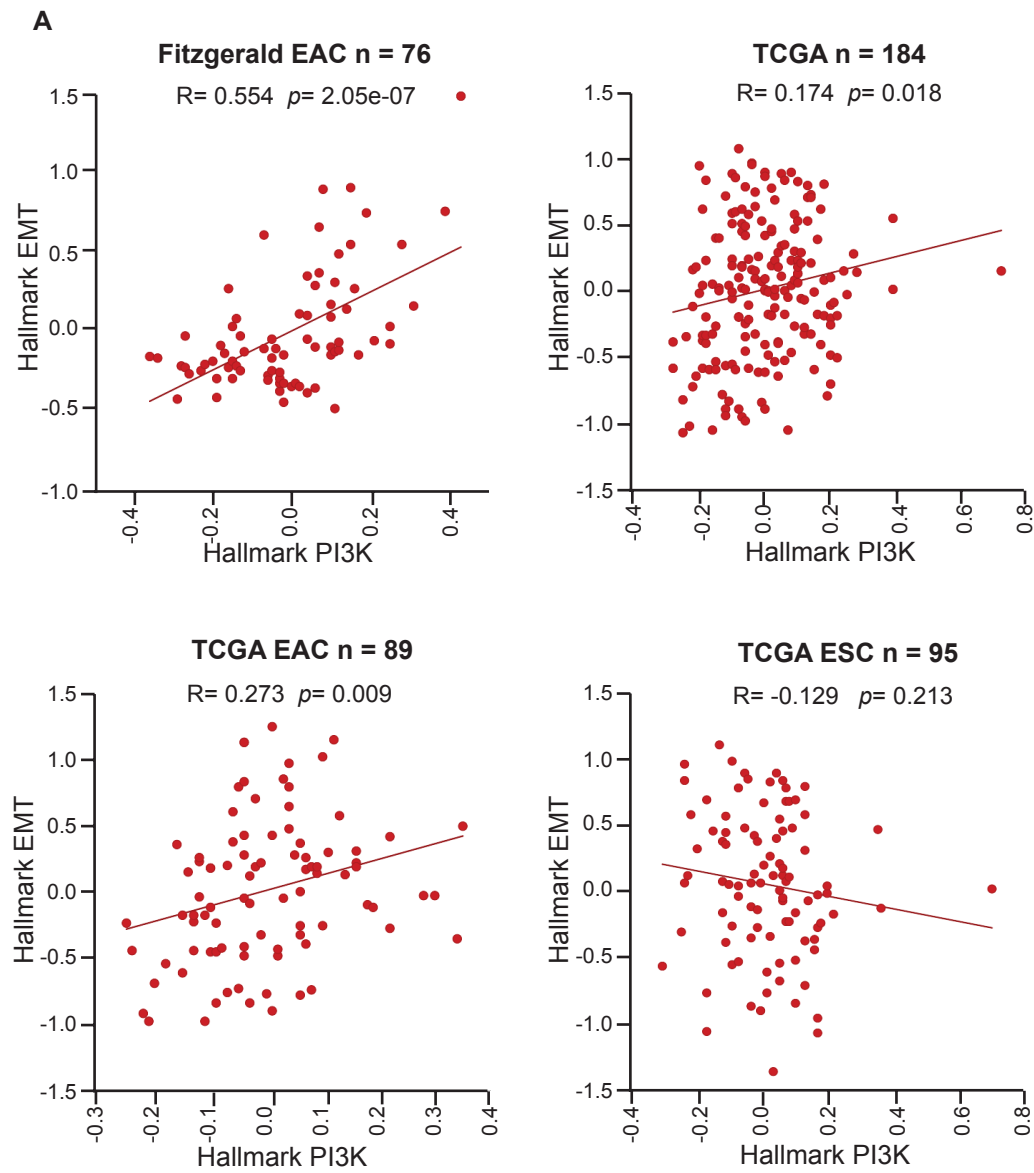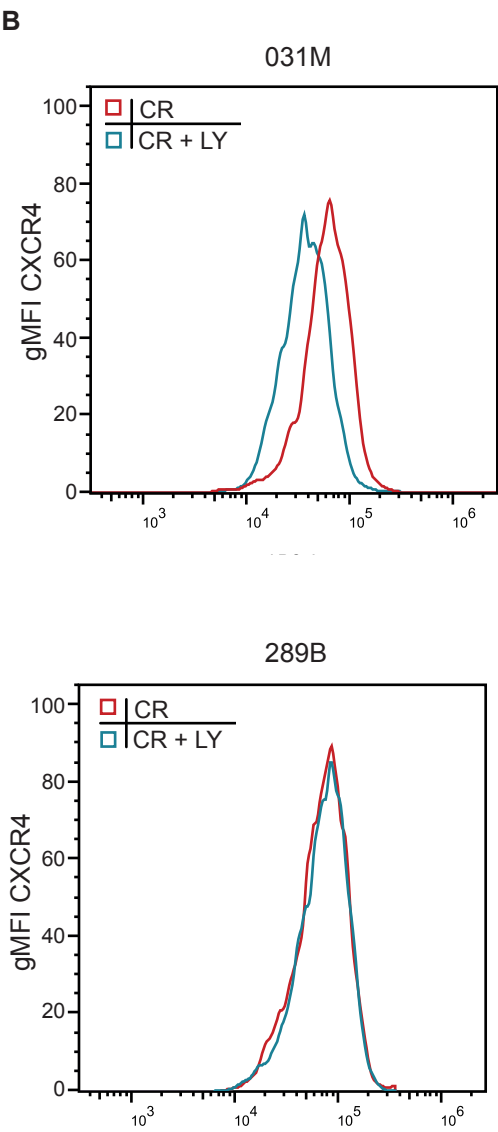

Supplement: Supplementary file 3 — Additional file 3: Figure S3. PI3K inhibitors can revert CRT-induced EMT. A Correlation of gene expression of Broad Hallmark Pi3K_AKT_mTor_signalling gene set with the Broad Hallmark Epithelial_Mesenchymal_transition gene set in two publicly available datasets, GSE96669 and Esophageal Carcinoma Tumor Cancer Genome Atlas (TCGA-ESCA), respectively. B FACS analyses of mesenchymal marker CXCR4 after 7 days of treatment with CRT with or without 500 nM LY3023414. 031 M poor CRT responder, 289B good CRT responder. gMFI = geometric mean fluorescent intensity. [file 12967_2022_3376_MOESM3_ESM.pdf]

Supplementary figure 4

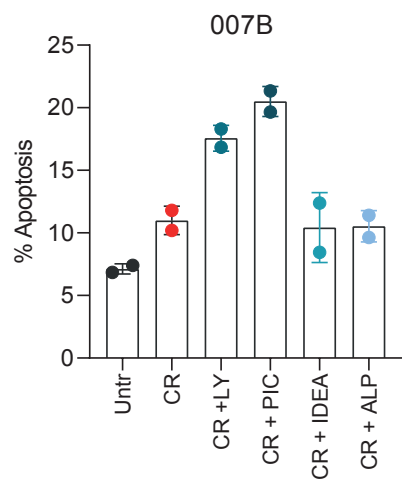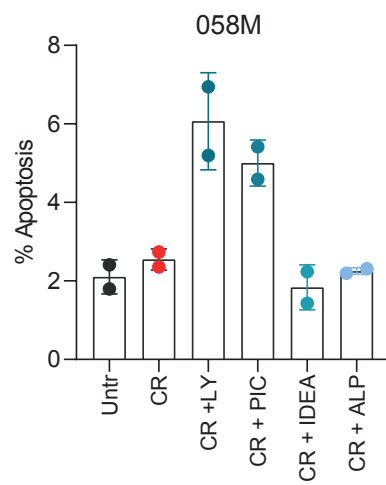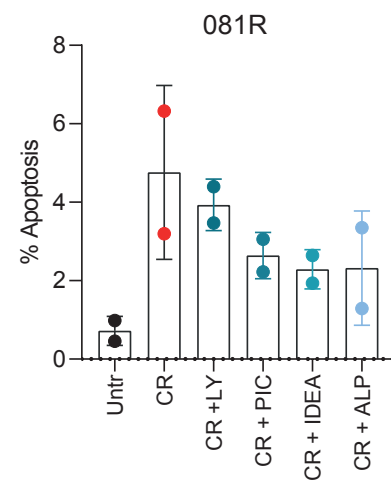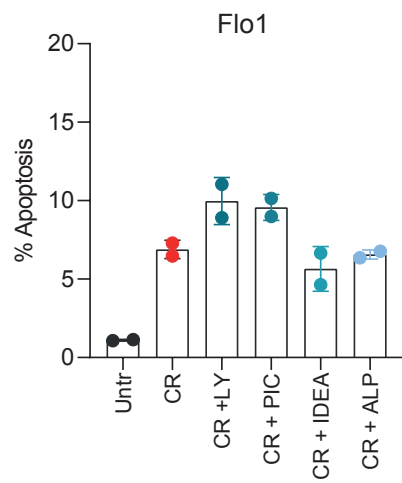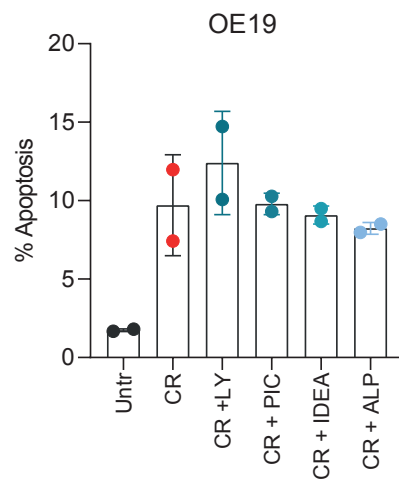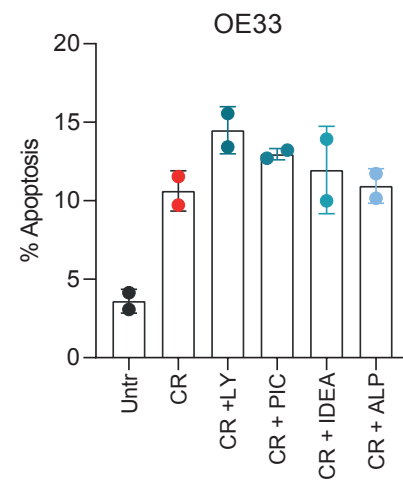

Supplement: Supplementary file 4 — Additional file 4: Figure S4. Apoptosis induced by PI3K pathway inhibitors in poor responder CRT cell lines. Poor CRT responder cell lines 007B, 058 M and 081R and good responder cell lines Flo1, OE19 and OE33 were treated for 7 days with the CRT regimen in addition to 500 nM of PI3K pathway inhibitors (based on average IC50 of four compounds). Apoptosis measured by percentage of green fluorescent Annexin V-FITC. Data points represent biological replicates, mean with SD. [file 12967_2022_3376_MOESM4_ESM.pdf]
