# Supplementary material for: FOXO transcriptional activity is associated with response to chemoradiation in EAC
Source: J Transl Med. 2022 Apr 25;20:183. doi: 10.1186/s12967-022-03376-w (PMC9036728; doi:10.1186/s12967-022-03376-w)
Supplement: Supplementary file 5 — Additional file 5: Table S1. Characteristics of the five established patient derived cell lines. Table S2. FDA approved PI3K inhibitors tested in this study. Table S3 Western blot antibodies used in this study. [file 12967_2022_3376_MOESM5_ESM.docx]

**SUPPLEMENTARY METHODS**

**Western blot**

Pre-treatment cells were lysed in RIPA buffer (Cell Signaling) containing phosphatase and protease inhibitor cocktail (Cell Signaling). Protein levels were determined by BCA (Pierce). Samples were heated for 5 min at 95 ºC loaded on 4-20% polyacrylamide precast gels (Bio-Rad) and transferred to PVDF membranes. Samples were blocked with 5% BSA (Lonza) in Tris buffered saline with 0.1% Tween-20 (TBS-T), and incubated overnight at 4°C with primary antibodies (Supplementary table S3). All were used at 1:1000. (HRP)-conjugated secondary were used at 1:5000 and incubated for 2 hours at room temperature. Proteins were imaged using a FuijFilm LAS 4000 imager (Fuji), using ECL Western blotting substrate (Pierce). Western blot bands were quantified using Image J by dividing protein of interest per lane by the housekeeping protein.

**Cell viability assay**

Cell viability was determined using a Cell Titer-Blue Cell Viability Assay kit (G8081; Promega, Madison, WI). Cells were seeded into 96-well plates in triplicates. After cell adhesion overnight, cells were treated. After one week, cell viability was measured by adding 20 μL of Cell Titer-Blue reagent to each well followed by three hours incubation. Plates were read at 560/590nm in a cytofluormeter (BioTek Instruments). Viability was calculated from values from CRT cells with or without PI3K inhibitors, minus baseline cell viability.

**Imaging based proliferation assay**

Proliferation was determined using IncuCyte™ live cell imaging system (Essen BioScience), quantitatively detecting live cells. Cells were imaged after one week of treatment.

**Apoptosis assay**

Apoptosis was similarly assessed using the IncuCyte system, by incubating cells in 0.33 mg/mL annexin V-FITC, administered simultaneously with drugs. Cells were imaged after one week of treatment. Apoptotic fraction was calculated by the ratio of FITC-positive cell area, divided by confluence (total cell area).

**Gene expression database analysis**

Gene expression of Broad Hallmark *PI3K_AKT_mTor_signaling* gene set was correlated with the Broad Hallmark *Epithelial_Mesenchymal_transition* gene set in two publicly available datasets: Esophageal Adenocarcinoma Fitzgerald (GSE96669)(17) and Esophageal Carcinoma Tumor Cancer Genome Atlas (TCGA-ESCA; https://gdc-portal.nci.nih.gov/projects/TCGA-ESCA)(4). Analysis was performed using the web‐based genomics platform R2 (R2: Genomics Analysis and Visualization Platform, http://r2.amc.nl).

**Statistical analyses**

Pathway activity between normal and EAC tissue were calculated using the Mann-Whitney test to compare ranks. In case of paired samples - between matched pre-treatment biopsies and resection specimen or resection specimen and recurrence, the Wilcoxon matched-pairs signed ranked test was used. Correlation of pathway activities was assessed using Pearson’s correlations. Sensitivity analyses were performed for patients receiving panitumumab(9). Survival analyses were performed using Kaplan-Meier and multivariable Cox proportional hazard regression analysis, including clinically relevant clinicopathological variables. Statistical analyses were performed in R. A p-value of p<0.05 was regarded statistically significant. In all *in vitro* experiments Spearman correlation tests were performed using GraphPad Prism 8. Error bars in bar graphs indicate the mean ± SD. A *p-*value of p<0.05 was considered statistically significant.

**SUPPLEMENTAL LEGENDS**

**Supplementary Figure 1 | Poor responder phenotype in patient samples.**

A) Pathway signal transduction activity of six key signal transduction pathways was measured in the resectable disease cohort (i). Pre-treatment biopsy pathway activity scores were subtracted from all matched resections that both passed QC, i.e. delta activity score (N=69). Two-sided Wilcoxon signed-rank statistical tests were performed between all post-nCRT (N=56) and surgery only (N=13) patients. *p-*values are indicated in the figures. Boxplots represent median with interquartile range.

B) All post-nCRT resection specimens from the resectable disease cohort were assessed for correlations between FOXO and TGF-β activity. Spearman correlations were performed, N=138, separated for low (1-2, N=17, all Mandard 2), middle (3, N=59) and high (4-5, N=47) Mandard score.

C) Pre-treatment biopsies were assessed for correlations between FOXO and TGF-β activity. Spearman correlations were performed, N=77, separated for low (1-2, N=22), middle (3, N=37) and high (4-5, N=18) Mandard score obtained after nCRT.

D) Disease free survival of patients with combined low FOXO and TGF-β pathway activity versus combined high pathway activities in post-nCRT resection specimens (N=83). Cut-off by median pathway activity score.

**Supplementary Figure 2 | Validation of PI3K pathway inhibition and sensitization of poor CRT responder cells.**

A) Correlation of baseline FOXO transcriptional activity with PI3K pathway activity based on P-S6, P-AKT and P-ERK PI3K in all eight EAC cell lines.

B) Poor CRT responder cell line 031M and good CRT responder cell line 289B were exposed for 7 days to the CRT regimen in combination with 500nM LY3023414, Alpelisib, Pictilisib or Idelalisib. Cells were lysed on day 8. Western blot analysis of PI3K-FOXO pathway by P-AKT and P-S6K as proteins of interest, β-actin as loading control.

C) Quantification of Western blot in A corrected for α-tubulin.

D) Cells were treated for 7 days with the CRT regimen, including a concentration range of 0, 62.5, 125, 250, 500 and 1000 nM Alpelisib, Idelalisib, Pictilisib or LY3023414. Percentage viable cells were measured on day 8 and plotted normalized to CRT. Data represents two biological replicates with SEM.

**Supplementary Figure 3 | PI3K inhibitors can revert CRT-induced EMT.**

A) Correlation of gene expression of Broad Hallmark *Pi3K_AKT_mTor_signalling* gene set with the Broad Hallmark *Epithelial_Mesenchymal_transition* gene set in two publicly available datasets, GSE96669 and Esophageal Carcinoma Tumor Cancer Genome Atlas (TCGA-ESCA), respectively.

B) FACS analyses of mesenchymal marker CXCR4 after 7 days of treatment with CRT with or without 500 nM LY3023414. 031M poor CRT responder, 289B good CRT responder. gMFI = geometric mean fluorescent intensity.

**Supplementary Figure 4 | Apoptosis induced by PI3K pathway inhibitors in poor responder CRT cell lines.**

Poor CRT responder cell lines 007B, 058M and 081R and good responder cell lines Flo1, OE19 and OE33 were treated for 7 days with the CRT regimen in addition to 500 nM of PI3K pathway inhibitors (based on average IC50 of four compounds). Apoptosis measured by percentage of green fluorescent Annexin V-FITC. Data points represent biological replicates, mean with SD.

**SUPPLEMENTAL TABLES**

**Supplemental Table S1** | Characteristics of the five established patient derived cell lines.
GEJ: Gastroesophageal junction, CROSS: ChemoRadiotherapy for Oesophageal cancer followed by Surgery Study, CapOX: CAPecitabine and Oxaliplatin, dCRT: definitive ChemoRadioTherapy. Res: Resection.

|  | |  |  |  |  |  |  |  |  |
| --- | --- | --- | --- | --- | --- | --- | --- | --- | --- |
| **Patient number** | **Gender** | **Survival** | **Origin cell line** | **Subtype** | **Age at diagnosis** | **BMI** | **HER2 status** | **cTNM** | **Differentiation grade** |
| AMC-007-EAC | F | Alive | Biopsy | EAC | 50 | 21,7 | negative | cT3N1M0 | 2 |
| AMC-031-EAC | M | Deceased | Metastases | EAC | 78 | 24,8 | negative | cT3N3M1 | 3 |
| AMC-058-EAC | M | Deceased | Metastases | EAC | 76 | 27,5 | negative | cT3N2M0 | NA |
| AMC-081-EAC | M | Deceased | Resection | EAC | 38 | 24,7 | negative | cT3N1M1 | 3 |
| AMC-289-EAC | M | Deceased | Biopsy | EAC | 51 | 31,7 | negative | cT3N0M0 | 3 |
|  |  |  |  |  |  |  |  |  |  |
| **Patient number** | **Tumor location** | **Length tumor** | **Treatment after biopsy** | **Treament tissue** | **Resection yes no** | **Mandard** | **pTNM** | **Recurrence** | **Treatment of recurrence** |
| AMC-007-EAC | GEJ/cardia | 5 | CROSS | None | yes | 4 | pT3N1M0 | Yes | palliative |
| AMC-031-EAC | distal | 7 | CapOx | CapOx | no | NA | NA | Yes | NA |
| AMC-058-EAC | GEJ/cardia | 7 | CROSS | CROSS | no | NA | NA | Yes | palliative |
| AMC-081-EAC | distal | 7 | dCRT | dCRT +Res | yes | NA | NA | Yes | other |
| AMC-289-EAC | GEJ/cardia | 8 | nCT | None | no | NA | NA | Yes | palliative |
|  |  |  |  |  |  |  |  |  |  |

**Supplementary table S2 |** FDA approved PI3K inhibitors tested in this study

| **PI3K inhibitor** | **Compound name** | **Targets** | **IC50 literature** | **References** | **Status** |
| --- | --- | --- | --- | --- | --- |
| LY3023414 | LY3023414 | class I PI3K, mTOR, DNA-PK | 6.07 nM | (35); (36) | Phase 1, Preclin. EAC |
| Alpelisib | BYL719 | p110 alfa-isoform PI3K | 5 nM | (34); (37) | Phase 3 |
| Pictilisib | GDC-0941 | p110 alfa, delta isoform PI3K | 3 nM | (38); (39) | Phase 1b |
| Idelalisib | CAL-101(GS-1101) | p110 delta isoform PI3K | 2.5 nM | (40) | Phase 1b |

**Supplementary table S3 |** Western blot antibodies used in this study

| **Primary** | **Antibody** | **Cat nr** | **Dilution** | **Species** | **kDa** | **Company** |
| --- | --- | --- | --- | --- | --- | --- |
|  | Phospho-44/42 MAP Kinase (Thr202/Tyr204) | 9101 | 1:1000 | Rabbit | 42, 44 | Cell Signaling |
|  | Phospho-pAKT | 4060/D9E | 1:1000 | Rabbit | 60 | Cell Signaling |
|  | Phospho-p70 S6 Kinase (Thr389) | 2211S | 1:1000 | Rabbit | 32 | Cell Signaling |
|  | α-Tubulin | Sc-23948 | 1:1000 | Mouse | 55 | Santa Cruz |
| **Secondary** |  |  |  |  |  |  |
|  | HRP-conjugated Goat anti rabbit | 7074 | 1:5000 | Anti-rabbit | NA | Cell Signaling |
|  | HRP-conjugated Goat anti mouse | sc-2357 | 1:5000 | Anti-mouse | NA | Santa Cruz |
